# Identification of Single Nucleotide Polymorphisms Associated with Hyperproduction of Alpha-Toxin in *Staphylococcus aureus*

**DOI:** 10.1371/journal.pone.0018428

**Published:** 2011-04-08

**Authors:** Xudong Liang, Jeffrey W. Hall, Junshu Yang, Meiying Yan, Katherine Doll, Russell Bey, Yinduo Ji

**Affiliations:** Department of Veterinary and Biomedical Sciences, College of Veterinary Medicine, University of Minnesota, St. Paul, Minnesota, United States of America; Cairo University, Egypt

## Abstract

The virulence factor α-toxin (*hla*) is needed by *Staphylococcus aureus* in order to cause infections in both animals and humans. Although the complicated regulation of *hla* expression has been well studied in human *S. aureus* isolates, the mechanisms of of *hla* regulation in bovine *S. aureus* isolates remain undefined. In this study, we found that many bovine *S. aureus* isolates, including the RF122 strain, generate dramatic amounts of α-toxin *in vitro* compared with human clinical *S. aureus* isolates, including MRSA WCUH29 and MRSA USA300. To elucidate potential regulatory mechanisms, we analyzed the *hla* promoter regions and identified predominant single nucleotide polymorphisms (SNPs) at positions −376, −483, and −484 from the start codon in α-toxin hyper-producing isolates. Using site-directed mutagenesis and *hla* promoter-*gfp*-*luxABCDE* dual reporter approaches, we demonstrated that the SNPs contribute to the differential control of *hla* expression among bovine and human *S. aureus* isolates. Using a DNA affinity assay, gel-shift assays and a null mutant, we identified and revealed that an *hla* positive regulator, SarZ, contributes to the involvement of the SNPs in mediating *hla* expression. In addition, we found that the bovine *S. aureus* isolate RF122 exhibits higher transcription levels of *hla* positive regulators, including *agrA*, *saeR*, *arlR* and *sarZ*, but a lower expression level of *hla* repressor *rot* compared to the human *S. aureus* isolate WCUH29. Our results indicate α-toxin hyperproduction in bovine *S. aureus* is a multifactorial process, influenced at both the genomic and transcriptional levels. Moreover, the identification of predominant SNPs in the *hla* promoter region may provide a novel method for genotyping the *S. aureus* isolates.

## Introduction

*S. aureus* is an important pathogen capable of causing both animal and human infections such as pneumonia, endocarditis, toxic shock syndrome and bovine mastitis. The continuous increase of infections associated with both hospital- and community-acquired methicillin resistant *Staphylococcus aureus* (HA-MRSA and CA-MRSA) has caused serious public health concerns [1]. The ability of this organism to cause a wide variety of infections partially depends on the coordinated and regulated expression of multiple cell- and surface-associated virulence factors [2], [3] and exported proteins including various proteases and toxins [4].

Alpha-toxin plays a critical role in the modulation of *S. aureus*–induced cytotoxicity in Jurkat T-lymphocytes, human peripheral blood lymphocytes, monocytes [5] and epithelial cells [6], even though multiple virulence factors are required for the bacterium to induce apoptosis in endothelial cells [7]. Alpha-toxin can interact specifically with surface receptors of the host cells, form functional transmembrane pores, and selectively release ions, and/or leads to the activation of cell signaling pathways, thus inducing apoptosis and/or necrosis in various cell types [8]–[12]. Recently, we demonstrated that α-toxin interacts with α5β1-integrin to interfere with *S. aureus* adhering to and internalizing into human lung epithelial cells (A549) [13]. The interaction of α-toxin with α5β1-integrin contributes to the cytotoxicity of α-toxin that is required for *S. aureus* to induce apoptosis and death of the epithelial cells [6]. However, the role played by α-toxin depends on the stage and/or type of infection and the quantities produced. It has been demonstrated that α-toxin is an important virulence factor in experimental brain abscesses and pneumonia [14]–[16] and intraperitoneal infection [17], [18], whereas the overproduction of α-toxin significantly reduces virulence in experimental endocarditis [19].

Alpha-toxin expression is up-regulated in the stationary growth phase *in vitro* [20] and at later stages of animal infection [21], [22]. The expression of α-toxin is simultaneously regulated by different regulators, which have been well documented and reviewed elsewhere [20], [23]. The expression of α-toxin is positively regulated by various global regulators, including two-component signal transduction systems, such as the accessory gene regulator (Agr) [24], [20], the staphylococcal accessory protein effector (SaeRS) [25], [26], ArlRS [27], [28] and transcriptional regulators Mgr [29] and SarZ [30]–[32]. In contrast, the homologues of staphylococcal accessory regulator (SarA), including Rot and SarT, repress the expression of α-toxin [33], [34]. The role of SarA in modulating *hla* transcription is controversial: SarA affects *hla* expression in both an *agr*-dependent and *agr*-independent manner [22], [35]. In addition, it has been revealed that *hla* transcription is affected by additional factors, including the alternative sigma B factor (σ^B^) and environmental stimuli [36], [37].

Alpha-toxin also serves as a virulence factor in *S. aureus*-induced mastitis [38], [39]. It has been found that significant increases in milk antibodies to α- and β-toxins are present in cows with chronic staphylococcal mastitis [40], and that many *S. aureus* isolates from the mammary gland of dairy cows produce α-toxin [41]. The comparative genomic analysis of human and bovine *S. aureus* isolates suggests that a unique mechanism may be involved in the pathogenicity of bovine *S. aureus* isolates [42] and that some bovine *S. aureus* isolates generate dramatic amounts of α-toxin [43]. However, the mechanism of hyperproduction of α-toxin in some bovine *S. aureus* isolates remains undefined.

In the present study, we compared the expression profiles of exported proteins between bovine *S. aureus* isolates and human clinical *S. aureus* isolates. We found hyperproduction of α-toxin in many bovine *S. aureus* isolates and investigated potential mechanisms of up-regulation of *hla* using site-direct mutagenesis, transcriptional promoter-reporter fusion, and quantitative RT-PCR approaches. Our results indicate α-toxin hyperproduction in bovine *S. aureus* is a multifactorial process, influenced at both the genomic and transcriptional levels.

## Results

### Hyperproduction of α-toxin in bovine mastitis *S. aureus* isolates, including RF122

**Figure 1:**
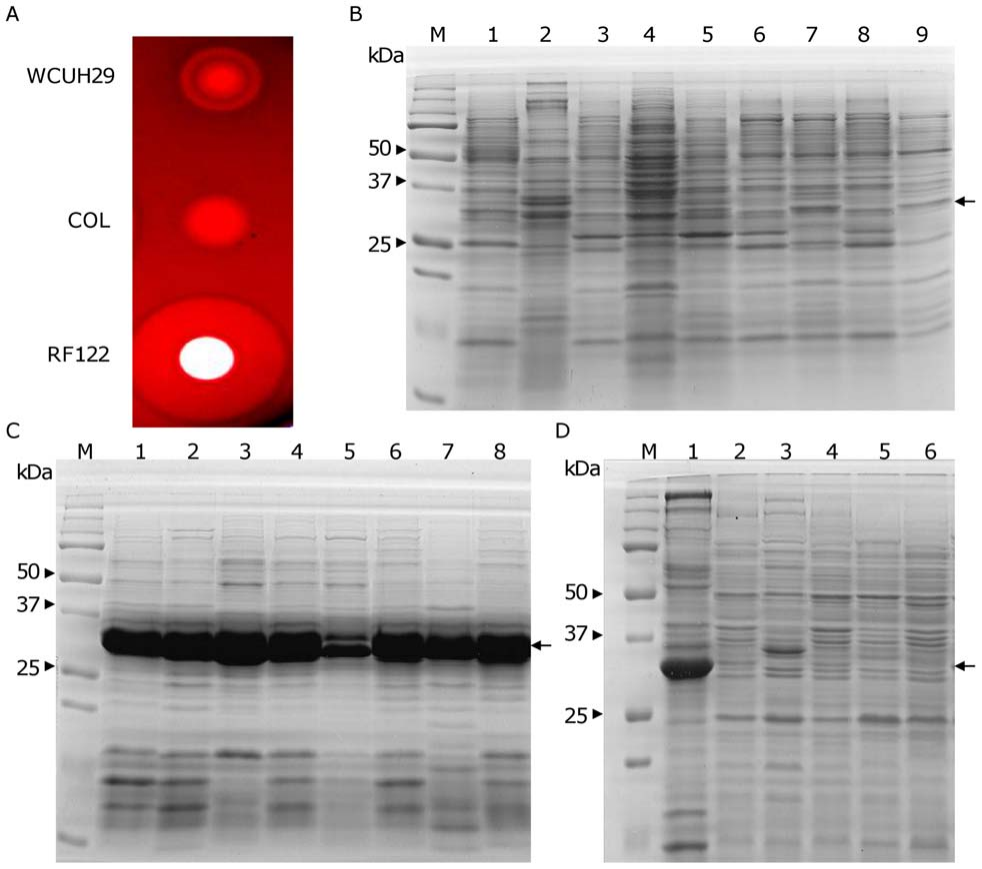
Hemolytic analysis and SDS-PAGE analysis of the expression profiles of exported proteins. (A) Hemolytic analysis on Sheep Blood Agar. SDS-PAGE analysis of the expression profiles of the exported proteins. (B) Human *S. aureus* isolates, Lane 1, WCUH29; Lane 2, NRS105; Lane 3, NRS194; Lane 4, NRS 237; Lane 5, NRS243; Lane 6, NRS248; Lane 7, NRS384; Lane 8, MW2(956); Lane 9, USA300(1371); M, Precision Plus Protein Standard. (C) Alpha-toxin hyper-producing bovine mastitis *S. aureus* isolates. Lane 1, RF122; Lane 2, BSa39; Lane 3, BSa55; Lane 4, BSa60; Lane 5, BSa67; Lane 6, BSa68; Lane 7, BSa74; Lane 8, BSa97. M, Precision Plus Protein Standard. (D) Bovine mastitis *S. aureus* isolates. Lane 1, RF122; Lane 2, BSa12; Lane 3, BSa22; Lane 4, BSa28; Lane 5, BSa83; Lane 6, BSa110. M, Precision Plus Protein Standard. Arrow indicates α-toxin.

The comparative genomic analysis of human and bovine *S. aureus* isolates suggested that a unique mechanism may be involved in the pathogenicity of bovine *S. aureus* isolates [42]. Numerous studies have demonstrated that staphylococcal exported proteins, especially toxins, are important virulence factors in bovine mastitis [40], [41]. To examine whether human and bovine *S. aureus* isolates produce distinct levels of toxins, we conducted hemolytic assays using a sheep blood agar plate. The supernatants from the overnight cultures of human isolates, WCUH29 and COL strains, had dramatically less hemolytic activity compared to that of bovine isolate RF122 (Fig. 1A). To further determine whether there are different exported protein profiles between human and bovine *S. aureus* isolates, we chose human clinical isolates, including WCUH29, different strains of MRSA and twelve bovine *S. aureus* isolates, including the RF122 strain, to compare their exported proteins using SDS-PAGE. None of the human isolates produced dramatic amounts of α-toxin (Fig. 1B). In contrast, seven bovine strains, including RF122, exhibited extremely high levels of α-toxin production (Fig. 1C), although five bovine strains produced α-toxin at levels similar to the human isolates (Fig. 1D). To further confirm the identity of the over-expressed protein, we conducted a MALDI mass spectrometry assay and identified that the highly expressed protein is α-toxin (data not shown). Furthermore, qPCR analysis identified that RF122 has a 7-fold higher level of *hla* transcript compared to WCUH29, suggesting the up-regulation of α-toxin is at the transcription level (Table 1).

**Table 1:** Comparison of gene expression by qPCR analysis between RF122 and WCUH29 strain.

ORF (N315)	Gene	Fold change (RF122/WCUH29)
Sa1007	*hla*	+7±0.1**
Sa2174	*sarZ*	+2±0.6
Sa1246	*arlS*	+2±0.1**
Sa0660	*saeS*	+5
Sa1844	*agrA*	+26±0.1**
Sa1583	*rot*	−2±0.1*
16SrRNA		0

*P≤0.05;

**P≤0.01.

### Identification of single nucleotide polymorphisms (SNPs) in the *hla* promoter region

**Figure 2:**
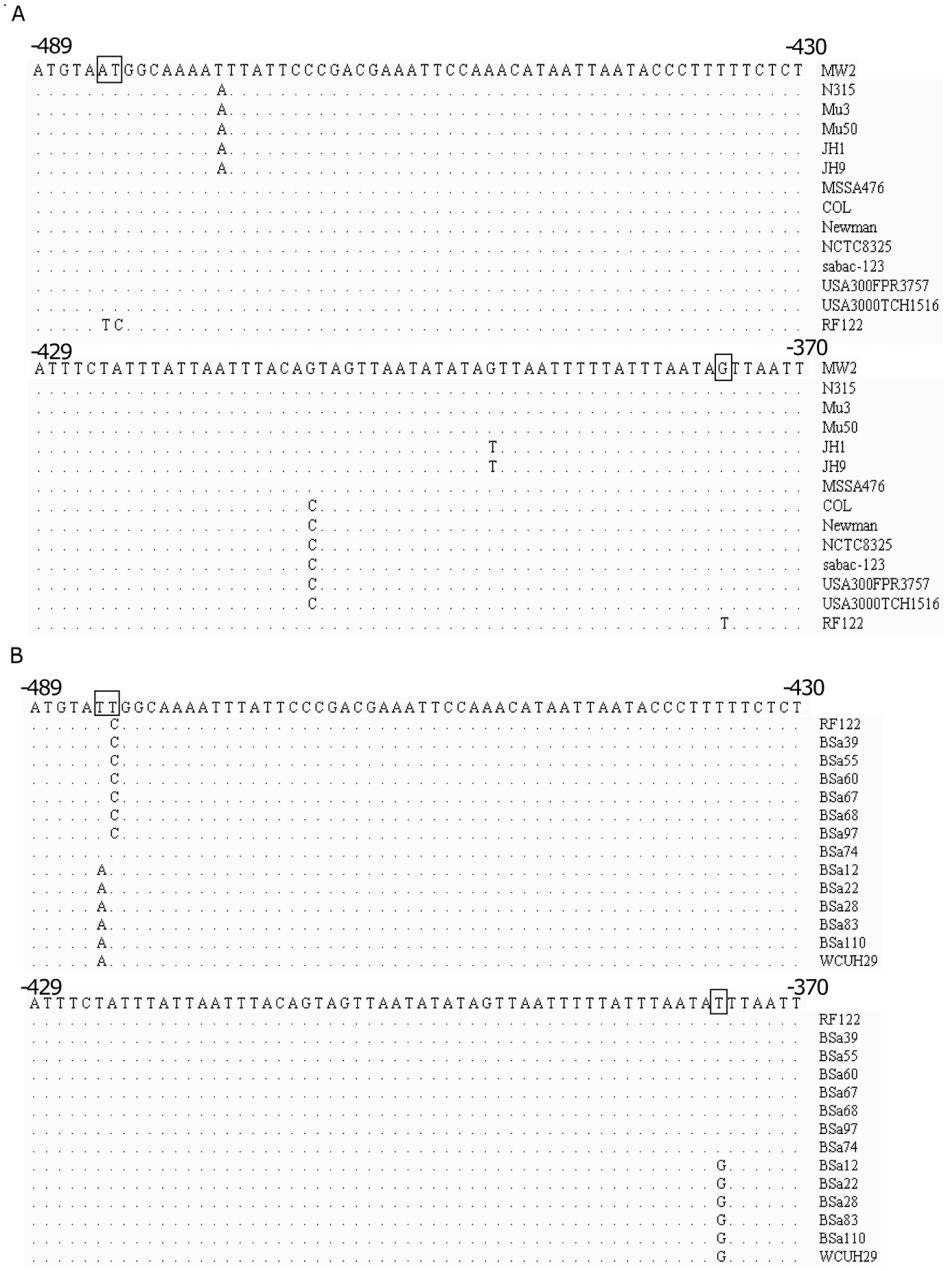
Structural alignments of Bovine and Human *S. aureus* isolates. Structural alignments with homologues of *hla* promoter region from sequenced bovine and published human *S. aureus* genomes. The symbol “–” represents the upstream region from the start codon of *hla*. The boxed nucleotide represents the major difference between bovine *S. aureus* isolates and the human *S. aureus* isolate. (A) Human *S. aureus* isolates (B) Bovine mastitis *S. aureus* isolates.

To elucidate the potential mechanisms involved in up-regulating *hla* expression in the bovine *S. aureus* isolate RF122, we performed alignment analyses of *hla* promoter region based upon the published *S. aureus* genomes in the NCBI genome database. We found that the DNA sequences of *hla* promoter region are almost identical among the human *S. aureus* isolates (Fig. 2A). However, several nucleotides of the *hla* promoter region of bovine RF122 in the positions −484, −483 and −376 from the start codon are different from those of human isolates (Fig. 2A). To further investigate whether these differences exist in other human and bovine isolates, we isolated the chromosomal DNA from these isolates and the *hla* promoter region was amplified by PCR for sequencing. The DNA sequencing results showed that among seven α-toxin over-expressing *S. aureus* isolates, six isolates possess the same *hla* promoter DNA sequence as RF122 (Fig. 2B), indicating that these bovine isolates have predominant SNPs in the *hla* promoter region. In contrast, the *hla* promoter sequences of the five α-toxin hypoproduction strains were identical to the human *S. aureus* isolates (Fig. 2B). In addition, the DNA sequences of *hla* promoter region from eight human isolates were identical to the published human isolates (data not shown).

### The SNPs in the *hla* promoter region affects *hla* transcription

**Figure 3:**
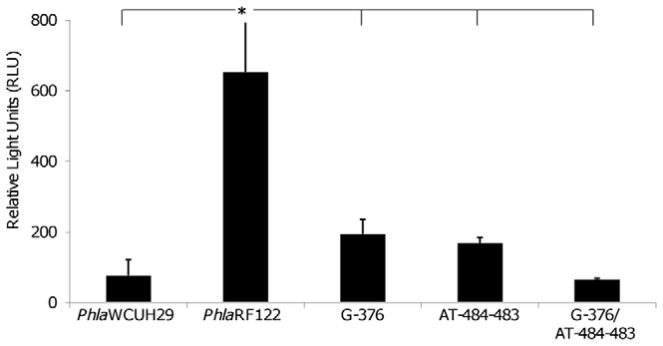
Influence of SNPs *hla* promoter-*luxABCDE* reporters on bioluminescence intensity of *S. aureus* WCUH29. The maximal light intensity values are given as relative light units (RLU). The symbol “*” indicates a significant difference (P≤0.05) between SaWH1207 and all other strains.

The identification of predominant SNPs in the *hla* promoter region led us to hypothesize that the SNPs may be associated with the modulation of *hla* transcription. To test this possibility, we created both the human and bovine *S. aureus hla* promoter-*gfp*-*luxABCDE* dual reporter constructs in the human isolate WCUH29 and determined the *hla* expression levels by measuring bioluminescence intensity. The dual reporter construct carrying the RF122 *hla* promoter showed a significantly higher level of *hla* transcription than the dual reporter construct carrying the WCUH29 *hla* promoter (Fig. 3). This data suggests that the predominant SNPs in the RF122 *hla* promoter region are likely involved in the transcriptional modulation of *hla* expression.

To confirm this finding, we first performed site-directed mutations in the *hla* promoter region using the RF122 *hla* promoter-*gfp*-*luxABCDE* dual reporter construct. The site mutations were confirmed by DNA sequencing. Then, we determined the impact of the nucleotide mutations on growth and *hla* expression by monitoring *lux* expression levels in the WCUH29 strain. The mutations in the *hla* promoter region had no impact on growth (data not shown). However, the nucleotide mutations in either −376 (T→G) or −484 and −483 (TC→AT) in the RF122 *hla* promoter region significantly decreased the reporter gene expression level compared to that of the parental promoter (Fig. 3). Furthermore, the double mutations (T→G/TC→AT) in the RF122 *hla* promoter region dramatically diminished the reporter gene expression, which was comparable to the construct carrying the WCUH29 *hla* promoter (Fig. 3).

**Figure 4:**
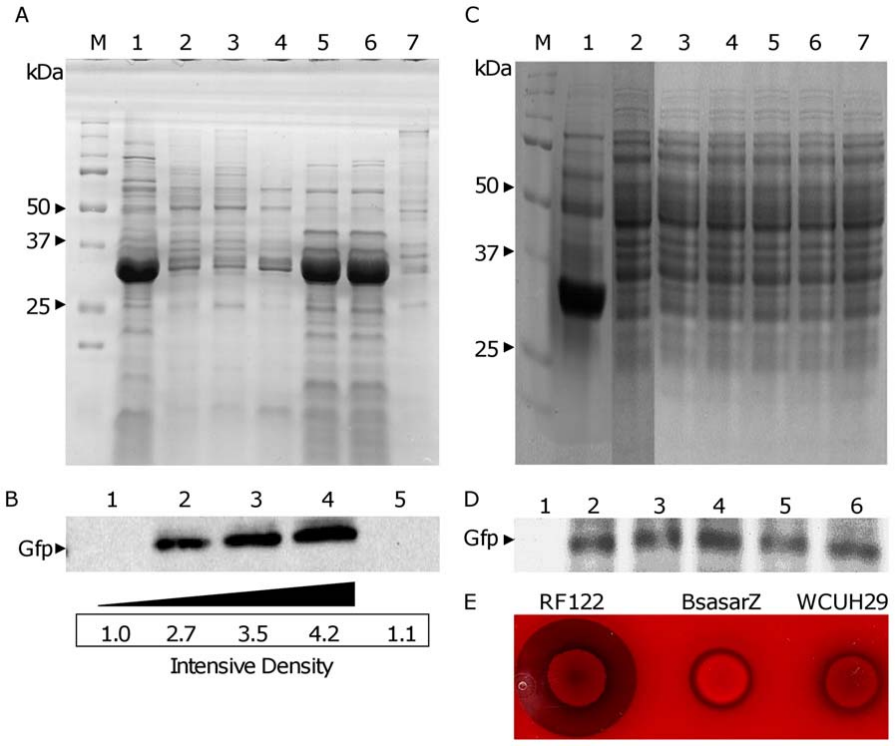
Impact of *hla* promoter and nucleotide mutations on *hla* expression in RF122 strain. (A) SDS-PAGE analysis of *hla* expression of the *S. aureus* RF122 strain carrying different *hla* promoter-*gfp* fusions. Lane 1, RF122 control; Lane 2, SaRF1207 (RF122 *hla* promoter-*gfp* fusion); Lane 3, SaRF1307 (G-376 mutated RF122 *hla* promoter-*gfp* fusion); Lane 4, SaRF1407 (AT-484-483 mutated RF122 *hla* promoter-*gfp* fusion); Lane 5, SaRF1507 (G-376 AT-484-483 mutated RF122 *hla* promoter-*gfp* fusion); Lane 6, SaRF1107 (WCUH29 *hla* promoter-*gfp* fusion); Lane 7, WCUH29 control. M, Precision Plus Protein Standard. (B) Western blot analysis of Gfp expression from different *hla* promoter-*gfp* fusions in RF122. Lane 1, SaRF1507; Lane 2, SaRF1307; Lane 3, SaRF1407; Lane 4, SaRF1207; Lane 5, SaRF1107. Black triangle indicates the increase of intensity of reaction band. (C) SDS-PAGE analysis of *hla* expression of the *S. aureus* RF122 *sarZ* null mutant (BsasarZ). Lane 1, RF122 control, Lane 2, BsasarZ control, Lane3, BSasarZ1107 (WCUH29 *hla* promoter-*gfp* fusion); Lane 4, BSasarZ1207 (RF122 *hla* promoter-*gfp* fusion); Lane 5, BSasarZ1307 (G-376 mutated RF122 *hla* promoter-*gfp* fusion); Lane 5, BSasarZ1407 (AT-484-483 mutated RF122 *hla* promoter-*gfp* fusion); Lane 7, BSasarZ1507 (G-376 AT-484-483 mutated RF122 *hla* promoter-*gfp* fusion); M, Precision Plus Protein Standard. (D) Western blot analysis of Gfp expression from different *hla* promoter-*gfp* fusions in BSasarZ. Lane 1, RF122 wild-type control; Lane 2, BSasarZ1107; Lane 3, BSasarZ1207; Lane 4, BSasarZ1307; Lane 5, BSasarZ1407; Lane 6, BSasarZ1507. (E) Hemolytic assay on Sheep Blood Agar.

To further confirm the role of the SNPs of the *hla* promoter region in controlling the *hla* over-expression in RF122 strain, we electroporated the above *hla* promoter-*gfp*-*lux* dual reporter constructs into the RF122 strain and examined the impact of the site-directed mutations in the *hla* promoter region on reporter gene expression. Surprisingly, no bioluminescence signal was detectable in the cultures of these strains. However, we found that the introduction of either the RF122 *hla* promoter-*gfp*-*lux* dual reporter or either of the single (T→G, TC→AT) mutated *hla* promoter-*gfp*-*lux* dual reporters into the RF122 strain either eliminated or severely reduced endogenous *hla* expression, respectively, indicating that dominant-negative effects occurred in the strains (Fig. 4A, lane 2, 3 and 4). In contrast, the introduction of the WCUH29 *hla* promoter-*gfp-lux* reporter and the double mutated (T→G/TC→AT) RF122 *hla* promoter-*gfp*-*lux* reporter did not have an appreciable effect on endogenous *hla* expression (Fig. 4A, lane 5 and 6). In addition, we determined the impact of the SNPs of *hla* promoter region on reporter *gfp* expression using a Western blotting assay. An intense band of Gfp was exhibited in the whole cell lysates of the reporter construct carrying the wild-type RF122 *hla* promoter; contrastingly, either no band or a weak band of Gfp was detected in the whole cell lysates of constructs carrying either the WCUH29 *hla* promoter or the double (T→G/TC→AT) mutated RF122 *hla* promoter (Fig. 4B). Both the T→G and the TC→AT mutations in the RF122 *hla* promoter led to a 2- to 3-fold decrease of *gfp* expression (Fig. 4B). Taken together, the above data demonstrate that the SNPs in the *hla* promoter region participate in the modulation of *hla* expression in the bovine *S. aureus* RF122 isolate.

### SarZ is associated with the SNPs in the regulation of hyperproduction of α-toxin

In order to identify regulators that are involved in the SNPs' regulation, we employed DNA affinity purification using a biotinylated RF122 *hla* promoter region oligonucleotide bound to Dynabeads M-280 Strepavidin coated paramagnetic beads. The cytoplasmic proteins of RF122 specifically binding to the *hla* promoter region oligonucleotide were eluted from the beads and separated by SDS-PAGE (data not shown). To determine the identity of *hla* promoter region binding proteins, we employed MALDI mass spectrometry assays and revealed SarZ and Mgr bound to the *hla* promoter region of RF122 (data not shown).

To confirm whether SarZ protein is associated with the SNPs in the modulation of *hla* expression genetically, we constructed a *sarZ* null mutant in the RF122 strain by phage transduction. The *sarZ* null mutant, BSasarZ, was confirmed by using diagnostic PCR and DNA sequencing. The five individual *hla* promoter-*gfp*-*lux* report vectors were introduced into BSasarZ by electroporation. First, we examined how the loss of SarZ affected the endogenous *hla* expression by using a Sheep Blood Agar hemolysis assay and SDS-PAGE and found that the *sarZ* null mutation abrogates the α-toxin hyper-production phenotype of RF122 (Fig. 4C, lanes 1 and 2; and E). Furthermore, the *sarZ* mutation eliminated the dominant-negative effects of the RF122 *hla* promoter-*gfp*-*lux* dual reporter (Fig. 4C, lanes 3 to 7) compared to the wild-type strains (Fig. 4A). Next, we determined the impact of SarZ on reporter *gfp* expression using a Western blotting assay. In contrast to the RF122 wild-type strain (Fig. 4B), no obvious difference of *gfp* expression was revealed in the whole cell lysates of *sarZ* mutants carrying the WCUH29 *hla* promoter, the single (T→G or TC→AT) mutated RF122 *hla* promoter, the double (T→G/TC→AT) mutated RF122 *hla* promoter, or the wild-type RF122 *hla* promoter (Fig. 4D).

To further confirm the specific effect of SarZ and identify binding affinity differences, we performed gel-shift assays. The PCR products were purified and labeled using DIG; the recombinant His-tagged SarZ protein was purified from *E. coli*. The gel-shift assays showed that with the addition of as little as 100 ng of SarZ protein a weak shifted band appeared for the RF122 *hla* promoter region, whereas no such band was detected for the WCUH29 *hla* promoter region (data not shown). These results suggest that the RF122 *hla* promoter binding affinity of SarZ is likely higher than the WCUH29 *hla* promoter region.

To examine whether the overproduction of α-toxin is also attributable to different levels of SarZ between human and bovine isolates, we performed real-time RT-PCR. We found that in the RF122 strain the transcriptional level of *sarZ* was 2-fold higher than that of the WCUH29 strain (Table 1). Taken together, the above results suggest that the SNPs' regulation of *hla* expression may function through SarZ directly. Furthermore, it suggests the high level of α-toxin in the RF122 strain is a result of an increased transcription level of *sarZ* and increased binding affinity for the RF122 *hla* promoter by SarZ, leading to an α-toxin hyper-producing phenotype. We did not investigate the impact of Mgr, because the DNA-binding motif (GTTG) of Mgr is outside the SNPs region in the *hla* promoter region [30].

### Involvement of *agr*, *arlRS*, *saeRS* and *rot* in the modulation of hyperproduction of α-toxin in the bovine *S. aureus* isolate RF122

It is well known that different regulators, including two-component signal regulators, such as Agr, SaeRS, and ArlRS systems, and transcriptional regulators such as SarA family proteins, such as Rot, coordinately regulate *hla* expression [20], [23]. Building off of our previous finding that RF122 has a higher *sarZ* transcript level, we hypothesized that the hyper-production of α-toxin may also be attributable to differential expression of these regulators between the bovine *S. aureus* RF122 and the human *S. aureus* WCUH29. To address this question, we examined the transcriptional levels of selected *hla* regulators using qPCR. The real-time RT-PCR results are shown in Table 1. In the bovine *S. aureus* RF122 strain, the transcriptional levels of *hla* positive regulator genes *agr*, *saeRS* and *arlRS* were higher than those in the human WCUH29 strain, whereas the transcriptional level of *hla* repressor gene *rot* was lower than that in the human WCUH29 strain (Table 1). This data suggests that the over-transcription of the *hla* positive regulator genes *agr*, *arlRS*, and *saeRS* and decreased transcription of the *hla* negative regulator gene *rot* also, at least in part, contributes to the α-toxin hyper-production phenotype of RF122.

## Discussion

Our studies revealed that the *hla* promoter region possesses predominant SNPs that contribute to the modulation of *hla* over-expression in some bovine *S. aureus* isolates. Although no specific SNP is identified in the *hla* promoter region of the human *S. aureus* isolates tested, we cannot exclude the possibility that SNPs may exist in the *hla* promoter region in other human *S. aureus* isolates. Notably, we identified that a known *hla* transcriptional regulator, SarZ, likely has a higher binding affinity for the SNPs region and contributes to the involvement of SNPs in regulating *hla* expression. Moreover, we revealed that the transcription level of *sarZ* is higher in the bovine *S. aureus* RF122 strain than in the human WCUH29 strain. This suggests that the higher transcription level of *sarZ* is, at least in part, involved in the α-toxin hyper-production phenotype of the RF122 strain. Importantly, we also demonstrated that the SNPs contribute to the regulation of *hla* regardless of the genetic background, WCUH29 or RF122, in that the RF122 *hla* promoter-*gfp*-*lux* dual reporter construct had higher levels of expression in both backgrounds compared to the WCUH29 *hla* promoter-*gfp-lux* dual reporter construct. Additionally, the site-directed mutation of the RF122 promoter's SNPs progressively decreased reporter expression in both backgrounds (Fig. 3 and 4B). Our findings are partially supported by a previous report that ST151 strains, including the RF122 strain, have elevated levels of *hla* and RNAIII expression [43]. Taken together, these findings indicate that unique mechanisms may be involved in the up-regulation of *hla* expression in bovine *S. aureus* isolates, which in turn may partially contribute to the pathogenicity in bovine mastitis.

DNA sequencing analysis indicates that there are predominant SNPs in the *hla* promoter region of bovine *S. aureus* isolates, which occur at positions −376, −483 and −484 upstream, of the *hla* start codon. In the α-toxin hyperproduction isolates, there are apparent nucleotide shifts from G to T and T to C at positions −376 and −483, respectively, compared with the α-toxin hypoproduction isolates. These nucleotide shifts lead to a decrease in the binding affinity of SarZ, which is consistent with the result of the *hla* promoter-*gfp* reporter assay. It has been reported that the DNA-binding protein, SarZ, regulates *hla* expression by binding to the *hla* promoter region [32], [31], and is associated with the pathogenicity of *S. aureus* [31]. Furthermore, the introduction of RF122 *hla* promoter-reporter fusion into the RF122 strain led to a dominant-negative effect on endogenous *hla* expression; this effect was eliminated in *sarZ* null background. Taken together, the above data indicate that the SarZ protein has a higher binding affinity for the RF122 *hla* promoter and is directly involved in differential regulation of *hla* expression through SNPs, which in turn may contribute to virulence of bovine *S. aureus*. Our results are similar to previous findings that a single nucleotide (T→C) mutation at position −215 bp in the promoter region of the nitrate reductase operon *narGHJI* in *Mycobacterium tuberculosis* and *Mycobacterium bovis* leads to differential activity of reductase and altered virulence capacity [44]; and that in *Vibrio cholerae*, nucleotide differences at positions −65 and −66 bp in the *tcpPH* (encoding a toxin-coregulated pilus transcriptional activator pair, TcpPH) promoter region is not only responsible for determining the classical and EL Tor biotypes, but also contributes to differential regulation of virulence gene expression through a *tcpPH* regulator, AphB [45]. Although we found that the DNA binding motif of Mgr is not located in the SNPs, we cannot exclude the possibility that Mgr may contribute to the hyperproduction of α-toxin, because *sarZ* gene is transcriptionally regulated by Mgr [32].

It was reported that the ET3 clone is predominant in bovine mastitis *S. aureus* isolates [46] and that different subtypes exhibit different expression levels of α-toxin in the ET3 clone [43]. The RF122 strain belongs to the ET3 clone ST151 subtype [43]; therefore, it is necessary to determine whether bovine *S. aureus* isolates that possess the predominant SNPs in the *hla* promoter region belong to the common bovine *S. aureus* clone (ET3) and/or the same subtype within the ET3 clone. The results would allow us to evaluate whether the identified predominant SNPs of the *hla* promoter region would be useful as a target for molecular diagnosis of *S. aureus* isolates that posses an α-toxin hyper-productive phenotype and may cause severe bovine mastitis.

The *agr* locus in *S. aureus* has been examined for its role in the expression of exotoxins and cell surface proteins [47]. It has been reported that variations in α-toxin production of *S. aureus* isolates from humans and bovines were due to variations of the RNA III transcript in the *agr* locus [8], [48]. Our data also revealed that in the RF122 strain the transcriptional levels of *hla* positive regulator genes *agr, saeR* and *arlR* are higher in the bovine *S. aureus* RF122 strain than in the human WCUH29 isolate in the same culture medium; however, the transcription level of the *hla* repressor gene *rot* is lower in the RF122 strain. Although the SNPs are located outside of the *hla* promoter DNA-binding regions (GEEAAN_6_GTTAA from −405 to −390 or TTTAAN_6_GTTA from −190 to −175) of phosphorylated SaeR [49] and the DNA-binding motif of Mgr [30], to define the mechanisms of their regulation is beyond the scope of this study. In addition, we found that the impact of SNPs on *hla* expression in the sigma B deficient strain RN4220 (Figure S1) is the same as in the sigma B positive strains WCUH29 and RF122. Thus, we can exclude the potential effect of sigma B factor on the SNPs' involvement in regulating *hla* expression, although sigma B factor is associated with mediating *hla* expression [36], [37].

It was previously reported that the RF122 strain belongs to ST151 (a subtype of ET3 clone) [43], [42]. Our unpublished data showed that the supernatant of RF122 culture is more toxic than the supernatant of WCUH29 using MAC-T cells [50]. This is consistent with recent findings that among ET3 subtypes, the ST151 strain is more virulent in an intramammary gland infection [43]. Previous studies have also demonstrated that most *S. aureus* isolates from the mammary gland of dairy cows produce α-toxin [41]. Significantly higher amounts of antibodies against both α- and β-toxins are exhibited in milk isolated from cows with chronic staphylococcal mastitis [40], and immunization of animals with attenuated α-toxin protects from *S. aureus*-induced mastitis [51]. Taken together, the above data suggest that the hyperproduction of α-toxin is a probable key factor for *S. aureus* to cause severe cow mastitis, although we cannot rule out the importance of other virulence factors in the pathogenesis of bovine mastitis [52].

In conclusion, we are the first to identify SNPs in the *hla* promoter region that results in the hyperproduction of α-toxin in many bovine *S. aureus* isolates. Importantly, we have identified and demonstrated that the DNA-binding protein SarZ contributes to the involvement of SNPs in differential regulation of *hla* expression. In addition, we found that the over-expression of *agrA*, *arlR* and *saeR*, and the down-regulation of *rot*, may be partially attributed to the hyperproduction of α-toxin in the RF122 strain.

## Materials and Methods

### Bacterial strains, plasmids and growth media

The bacterial strains and plasmids used in this study are listed in Table 2. The human *S. aureus* strains were obtained from the Network on Antimicrobial Resistance in *Staphylococcus aureus* or references indicated. The bovine *S. aureus* isolates were obtained from geographically diverse animals that received care at the University of Minnesota's Veterinary Diagnostics Laboratory. The *S. aureus* cells were cultured in Trypticase soy broth (TSB) at 37°C with shaking. *E. coli* strains were grown in Luria-Bertani (LB) medium. Transformants containing recombinant plasmids were selected on LB agar containing ampicillin (100 µg/ml) for *E. coli* and TSA containing chloramphenicol (10 µg/ml) for *S. aureus*.

**Table 2:** Strains and plasmids used in this study.

Strain /plasmid	Description	Reference or Resource
RN4220	laboratory *S. aureus* strain (*rsbU* ^−^)	[53]
WCUH29	human clinical MRSA isolate	NCIMB40771
COL	human clinical MRSA isolate	NARSA
NRS105–NRS384	human MRSA isolates	NARSA
MW2 and USA300	human MRSA isolates	[54]
RF122	bovine mastitis S. aureus isolate	[42]
BSasarZ	sarZ mutant of RF122 with sarZ::ermC	This study
BSa12–Bsa110	bovine mastitis S. aureus isolates	CVM Dia. Lab.
SaRN1107	RN4220 carrying pXL1107	This study
SaRN1207	RN4220 carrying pXL1207	This study
SaRN1307	RN4220 carrying pXL1307	This study
SaRN1407	RN4220 carrying pXL147	This study
SaRN1507	RN4220 carrying pXL1507	This study
SaWH1107	WCUH29 carrying pXL1107	This study
SaWH1207	WCUH29 carrying pXL1207	This study
SaWH1307	WCUH29 carrying pXL1307	This study
SaWH1407	WCUH29 carrying pXL1407	This study
SaWH1507	WCUH29 carrying pXL1507	This study
SaRF1107	RF122 carrying pXL1107	This study
SaRF1207	RF122 carrying pXL1207	This study
SaRF1307	RF122 carrying pXL1307	This study
SaRF1407	RF122 carrying pXL1407	This study
SaRF1507	RF122 carrying pXL1507	This study
BSasarZ1107	BSasarZ carrying pXL1107	This study
BSasarZ1207	BSasarZ carrying pXL1207	This study
BSasarZ1307	BSasarZ carrying pXL1307	This study
BSasarZ1407	BSasarZ carrying pXL1407	This study
BSasarZ1507	BSasarZ carrying pXL1507	This study
pCY1006	shuttle vector,derives from pSB2019, CmR, AmpR	[25]
pXL1107	WCUH29 *hla* promoter-*gfp*-*lux* reporter, Cm^R^, Amp^R^	This study
pXL1207	RF122 *hla* promoter-*gfp*-*lux* reporter, Cm^R^, Amp^R^	This study
pXL1307	RF122 (T→G) hla promoter-gfp-lux reporter, CmR, AmpR	This study
pXL1407	RF122 (TC→AT) hla promoter-gfp-lux reporter, CmR, AmpR	This study
pXL1507	RF122 (T→G/TC→AT) hla promoter-gfp-lux reporter, CmR, AmpR	This study

### SDS-PAGE analysis of exported proteins

The supernatants were collected from the overnight cultures of *S. aureus* isolates in TSB medium by centrifugation at 3900×g. The exported proteins were precipitated from an equal volume of supernatant using ethanol as described [17]. The exported protein profiles were detected by a sodium dodecyl sulfate (SDS)-12% polyacrylamide gel electrophoresis (PAGE) and Coomassie Blue staining.

### Statistical analysis

Data are the means ± standard errors of the means from three experiments. The sysmbol “*” indicates a significant difference (P≤0.05) using an unpaired *t* test.

### Construction and detection of promoter*-gfp-lux* dual reporter fusions

In order to further confirm the transcriptional regulation of *hla* expression, we created *hla* promoter-*gfp*-*lux* dual reporter constructs as previously described [25]. The *gfp*-*lux* dual reporter fusion system was provided courtesy of Philip Hill [55]. An approximate 1 kb upstream region of *hla* was amplified from both WCUH29 chromosomal DNA and RF122 chromosomal DNA by PCR, respectively, using the primers listed in Table 3, digested with *EcoR*I and *Xma*I, and ligated into the upstream region of the promoterless *gfp*-*lux* of pCY1006, which was digested with the same enzymes. The resulting recombinant plasmids pXL1107 and pXL1207 were electroporated into *S. aureus* RN4220, then into both WCUH29 and RF122, resulting in *S. aureus* strains SaRN1107, SaRN1207, SaWH1107, SaWH1207, SaRF1107 and SaRF1207. The *lux* expression was monitored until early stationary phase in TSB with an appropriate antibiotic at 37°C with a Chiron luminometer. The relative light units (RLU) were calculated (bioluminescence intensity/optical density at 600 nm). For western blot analysis of Gfp expression in different *hla* promoter-*gfp* fusions in RF122, a 1∶100 dilution of an overnight culture was grown and the same number of bacterial cells was harvested from cultures at an optical density of 1 at 600 nm by centrifugation. The whole cell lysates were prepared, and the same volume of lystate was loaded on 12% SDS-PAGE and probed by rabbit anti-Gfp antiserum using western blot assay. The density of the reaction band in equal area was scanned and calculated using ImageJ software.

**Table 3:** Primers used in this study.

Name	sequence
Sa0660RT	For 5′-CATTGCTATTAGCGATGAAGGTATTGG-3′Rev 5′-CTGCTTACACTGATTTTTGCGTTATTTTGTTG-3′
Sa1246RT	For 5′-ATGATAACACAGTGAGAGTTGAACC-3′Rev 5′-CTAACCCTTTGAAATCTTGCGTTG-3′
Sa1583RT	For 5′-TCAGCGAGATTGAAAGCGAATAC-3′Rev 5′-CTGTCCATTTCTTTAAGCGTCATAG-3′
Sa1844RT	For 5′-GTGAAATTCGTAAGCATGACCCAGTTG-3′Rev 5′-TGTAAGCGTGTATGTGCAGTTTCTAAAC-3′
Sa2174RT	For 5′- TGGAACACTGACACCATTAC-3′Rev 5′- CTGATGCTTCTCGTTCTGAA-3′
Sa1007RT	For 5′-CAACTGATAAAAAAGTAGGCTGGAAAGTGAT-3′Rev 5′-CTGGTGAAAACCCTGAAGATAATAGAG-3′
16S rRNART	For 5′-CTGTGCACATCTTGACGGTA-3′Rev 5′-TCAGCGTCAGTTACAGACCA-3′
PhlaT-Gfor	5′-GTTAATTTTTATTTAATAGTTAATTAATTGATTTA-3′
PhlaT-Grev	5′-TAAATCAATTAATTAACTATTAAATAAAAATTAAC-3′
PhlaTC-ATfor	5′-GATATTTCTATGTAATGGCAAAATTTATTCCCG-3′
PhlaTC-ATrev	5′-CGGGAATAAATTTTGCCATTACATAGAAATATC-3′
PhlaFor*EcoR*I	5′-ATGAATTCTTTAATCCCATATCACATTT-3′
PhlaRev*Xma*I	5′-TACCCGGGTTTCATCATCCTTCTATTTT-3′
Phla217for	5′-BiosgGCCTCTAACTAAAAACCTAC-3′
Phlarev482	5′-GTAATCGATTACAATATAAAAATAC-3′
Sa2174NdeIfor1	5′-TTCATATGATGTATGTAGAAAACAGCTATC-3′
Sa1274ZXhoIrev	5′-TTCTCGAGCTTTCTGTCGGAATAGTC-3′

### Site-directed mutagenesis

To determine whether *hla* transcription is modulated by single nucleotide polymorphisms (SNPs) in the RF122 *hla* promoter, site-directed mutations were generated by PCR using the pXL1207 as a template and the QuikChange site-directed mutagenesis kit (Stratagene, La Jolla, CA) according to the manufacturer's instructions except that primer extension was allowed to continue for 8 min. The primers used for site-directed mutagenesis are listed in Table 3. The mutations were confirmed by DNA sequencing of the region containing the mutation. The reformed plasmids were designated pXL1307, pXL1407 and pXL1507, and electroporated into RN4220, then into WCUH29 and RF122. The resulting *S. aureus* strains were named SaRN1307, SaRN1407, SaRN1507, SaWH1307, SaWH1407, SaWH1507, SaRF1307, SaRF1407 and SaRF1507.

The chromosomal DNA from each bovine mastitis *S. aureus* isolate listed in Table 2 was purified and the promoter region of *hla* from each isolate was obtained by PCR using the same primer listed in Table 3. The PCR products were purified and sequenced; the DNA sequences were deposited in GenBank (accession # HQ592340 to HQ592346).

### DNA affinity purification of *hla* promoter region binding proteins

In order to identify regulators associated with SNPs, we utilized Dynabeads M-280 strepavidin coated paramagnetic beads (Invitrogen, Carlsbad, CA) to identify DNA-binding proteins according to the manufacturer's protocol. A 266 bp PCR fragment spanning the RF122 *hla* promoter SNP region was PCR amplified using the primers RF122-phla217for and phlarev482 listed in Table 3 and purified using a PCR cleanup kit (Promega, Madison, WI). Approximately 9 µg of purified biotinylated *hla* promoter region was mixed with the beads. The mixtures were incubated at room temperature for 30 minutes with occasional gentle mixing. The beads were washed and resuspended in Protein Binding Buffer (10 mM Tris–HCl pH 7.5, 50 mM NaCl, and 1 mM DTT), then mixed with 500 µg of total cytoplasmic protein of RF122. The reaction mixtures were incubated at room temperature for 30 minutes with gentle, occasional swirling. The extra protein supernatant was removed and the beads were washed with Protein Binding Buffer to remove nonspecific binding proteins before being resuspended in 28 µl of Elution Buffer (10 mM Tris–HCl pH 7.5, 10% glycerol, 1 M NaCl, and 1 mM DTT) and incubated at room temperature for 30 minutes with occasional vortexing. A portion of the cytoplasmic protein fraction, washes, and elutes from the beads were detect by SDS-PAGE and visualized by Coomassie Blue staining.

### Construction of *sarZ* null mutant in RF122 strain

Construction of *sarZ* null mutant in RF122 isolate was performed by phage transduction as described [32]. Dr. Adhar Manna provided us with a *sarZ* mutant of RN6390 (AM1090). A phage Ф80α lysate of AM1090 was prepared to infect bovine *S. aureus* isolate RF122 to create a *sarZ* null mutant of RF122, which was confirmed by diagnostic PCR and DNA sequencing of the flanking regions of *sarZ*. This null mutant was designated as BSasarZ. The different *hla*-promoter-*gfp-lux* reporter fusions pXL1107, pXL1207, pXL1307, pXL1407, and pXL1507, were each electroporated into BSasarZ. The resulting *S. aureus* strains were named BSasarZ1107, BSasarZ1207, BSasarZ1307, BSasarZ1407, and BSasarZ1507.

### Cloning, expression, and purification of recombinant SarZ protein

The *sarZ* coding region was obtained by PCR using *sarZ* specific primers (sarZNdeIfor1 and sarZXhoIrev) listed in Table 3 from *S. aureus* and cloned into *Nde*I and *Xho*I sites of the *E. coli* expression vector pET24b. The recombinant DNA (pET*sarZ*) was confirmed by using PCR and DNA sequencing and transformed into *E. coli* strain BL21. The transformants were incubated until mid-log phase (OD600 nm = ∼0.4); followed by induction of *sarZ* expression by adding IPTG (final concentration 1 mM). The His-tagged SarZ protein expression and purification were conducted as described [56]. The purity of purified His-tagged SarZ protein was evaluated in a 12% SDS-PAGE followed by Comassic Blue staining.

### Gel shift assays

The primers (Phla217for and Phlarev482) used for gel shift analysis are listed in Table 3. A 266 bp DNA fragment of *hla* promoter upstream region encompassing the SNPs was obtained by PCR using either RF122 chromosomal DNA or WCUH29 chromosomal DNA as a template. The amplified DNA fragments were purified and labeled with Digoxigenin using the DIG GEL Shift Kits (Roche, Indianapolis, IN) according to the manufacturer's protocol. The DNA-binding and electrophoresis were performed as described [32]. Briefly, the purified PCR products were labeled with Digoxigenin using terminal transferase (Roche, Indianapolis, IN). The interaction of SarZ with DNA was conducted in a 10 µl reaction mixture containing 0.03 pmol DIG-labeled DNA, 1 mg of poly-(dI–dC), 25 mM NaH_2_PO_4_ (pH 8.0), 50 mM NaCl, 2 mM MgCl_2_, 1 mM DTT, 10% glycerol and increasing amount of SarZ protein. An unlabeled DNA fragment of the promoter region as a specific competitor was added into the reaction with 100-fold excess to the labeled probe. After incubation at 25°C for 20 min, the reaction mixtures were analyzed by 5% native PAGE.

### RNA purification and quantitative RT-PCR analysis (qPCR)

Overnight cultures of *S. aureus* (WCUH29 and RF122) were incubated in TSB medium and grown to the mid-exponential phase of growth (OD_600 nm_∼0.5) at 37°C with shaking. Cells were harvested by centrifugation at 3900×g, and the RNA was isolated by the RNAPrep Kit (Promega, Madison, WI) according to the manufacturer's instructions. Contaminating DNA was removed with the TURBO DNA-*free* Kit (Ambion, Austin, TX) and the RNA yield was determined spectrophotometrically at 260 nm. The integrity of the purified RNA was analyzed by electrophoresis in 1.2% agarose-0.66 M formaldehyde gels. The 23S and 16S rRNA bands were clear without any obvious smearing patterns.

The first strand cDNA was synthesized using reverse transcriptase SuperScript III (Invitrogen, Carlsbad, CA). PCR reactions were set up in duplicate by using the SYBR Green PCR Master Mix (Bio-Rad, Hercules, CA); there were also controls using purified total RNA as the templates. Real-time sequence-specific detection and relative quantification were performed with the Stratagene Mx3000P Real Time PCR System. Relative quantification of the product was calculated using the Comparative CT method, as described for the Stratagene Mx3000P system. The housekeeping gene 16s rRNA was used as an endogenous control [25]. All samples were analyzed in duplicate, normalized against 16s rRNA gene expression, and statistically analyzed by Student's *t* test, using Microsoft Excel software. *P* values of ≤0.05 were considered significant.

## Supporting Information

Figure S1 Influence of SNPs *hla* promoter-*luxABCDE* reporters on bioluminescence intensity of *S. aureus* RN4220. The maximal light intensity values are given as relative light units (RLU). The symbol “*” indicates a significant difference (P≤0.05) between SaRN1207 and all other strains.
(TIF)
