# Supplementary figures and images for: Identification of Single Nucleotide Polymorphisms Associated with Hyperproduction of Alpha-Toxin in Staphylococcus aureus
Source: PLoS One. 2011 Apr 8;6(4):e18428. doi: 10.1371/journal.pone.0018428 (PMC3072997; doi:10.1371/journal.pone.0018428)

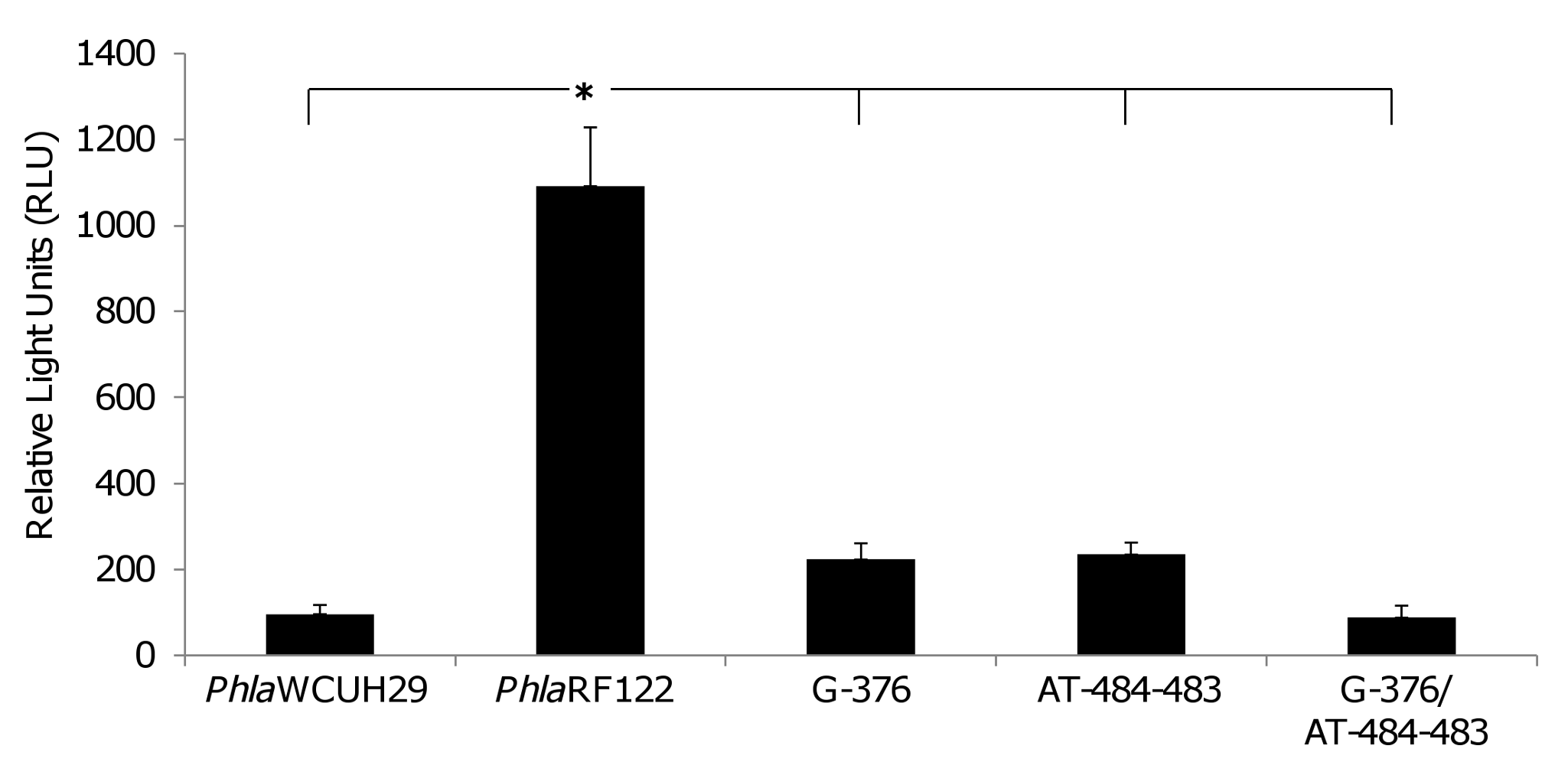

Supplement: Figure S1 — Influence of SNPs hla promoter-luxABCDE reporters on bioluminescence intensity of S. aureus RN4220. The maximal light intensity values are given as relative light units (RLU). The symbol “*” indicates a significant difference (P≤0.05) between SaRN1207 and all other strains. (TIF) [file pone.0018428.s001.tif]
